# Antimicrobial properties of chitosan from different developmental stages of the bioconverter insect *Hermetia illucens*

**DOI:** 10.1038/s41598-022-12150-3

**Published:** 2022-05-16

**Authors:** Anna Guarnieri, Micaela Triunfo, Carmen Scieuzo, Dolores Ianniciello, Elena Tafi, Thomas Hahn, Susanne Zibek, Rosanna Salvia, Angela De Bonis, Patrizia Falabella

**Affiliations:** 1 Department of Sciences, University of Basilicata, Potenza, Italy; 2 Spinoff XFLIES s.r.l, University of Basilicata, Potenza, Italy; 3 Fraunhofer Institute for Interfacial Engineering and Biotechnology IGB, Stuttgart, Germany

**Keywords:** Antimicrobials, Entomology

## Abstract

Growing antimicrobial resistance has prompted researchers to identify new natural molecules with antimicrobial potential. In this perspective, attention has been focused on biopolymers that could also be functional in the medical field. Chitin is the second most abundant biopolymer on Earth and with its deacetylated derivative, chitosan, has several applications in biomedical and pharmaceutical fields. Currently, the main source of chitin is the crustacean exoskeleton, but the growing demand for these polymers on the market has led to search for alternative sources. Among these, insects, and in particular the bioconverter *Hermetia illucens*, is one of the most bred. Chitin can be extracted from larvae, pupal exuviae and dead adults of *H. illucens*, by applying chemical methods, and converted into chitosan. Fourier-transformed infrared spectroscopy confirmed the identity of the chitosan produced from *H. illucens* and its structural similarity to commercial polymer. Recently, studies showed that chitosan has intrinsic antimicrobial activity. This is the first research that investigated the antibacterial activity of chitosan produced from the three developmental stages of *H. illucens* through qualitative and quantitative analysis, agar diffusion tests and microdilution assays, respectively. Our results showed the antimicrobial capacity of chitosan of *H. illucens,* opening new perspectives for its use in the biological area.

## Introduction

Nowadays, insect farming on an industrial scale has been increasingly developed, driven by two main issues: the search for new sources of protein for animal and human nutrition, and the need to dispose of an increasing amount of organic waste produced by farms and, more broadly, human activities^1,2^. The Black soldier fly, *Hermetia illucens* L. (Diptera: Stratiomyidae), has become the most important species for both feed production and waste management, being reared worldwide on a large scale and by 80% of all the European insect producers^3^.

*H. illucens* larvae can be grown on a wide variety of organic matter, of both vegetal and animal origin, and they are able to convert it into body mass rich in proteins and lipids^4–6^, used in feed, biofuel and cosmetic production^7–10^. Moreover, it is possible to obtain antimicrobial peptides, new molecules with great potential in pharmaceutical and biomedical fields^11–14^. The only by-products of *H. illucens* farming are dead flies and the exoskeleton derived from the moults made by the insect, as it moves from one developmental stage to the next during its life cycle. After hatching, *H. illucens* develops through five larval instars until the prepupal stage. Then, pupation occurs and, once the intra-puparial development is completed, the adult fly emerges. Shedding is constituted mainly by larval exoskeletons, pupal exuviae and, in addition, adult carcasses. Indeed, after mating and egg oviposition, the adults quickly die. All these by-products are rich in chitin^6,15^.

**Figure 1:**
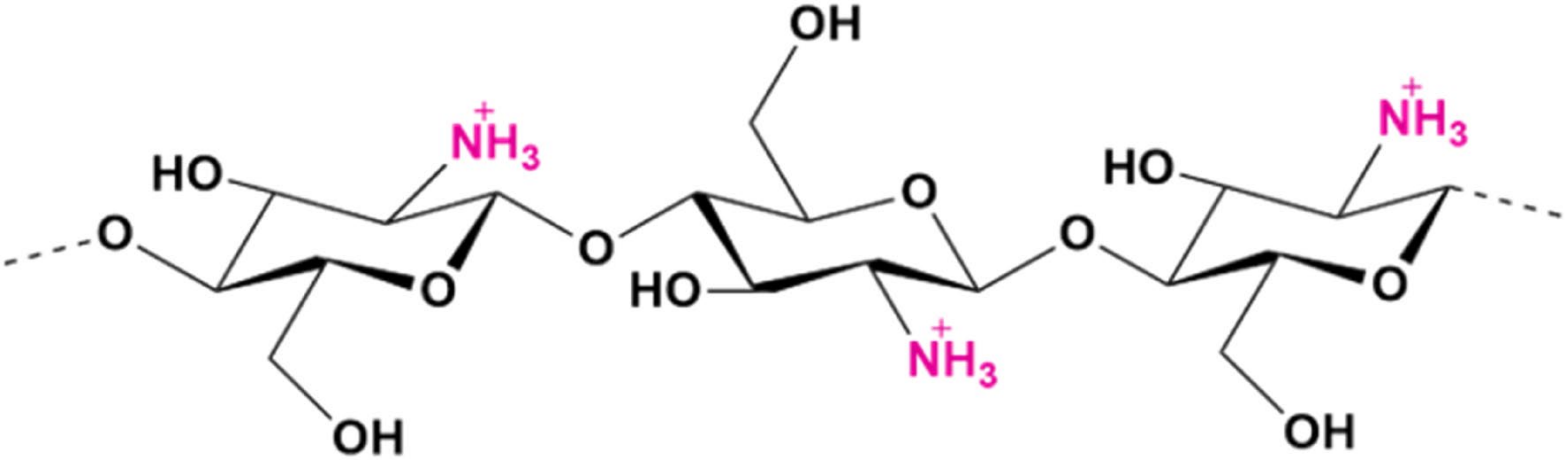
Structure of chitosan with its active amino groups, after their protonation in acid conditions, responsible for the antimicrobial activity (image obtained with ChemDraw).

Chitin and its derivatives are among the most important and exploited biopolymers for a wide range of applications^16^. Chitin, composed of β-(1,4)-linked N-acetyl- β-D-glucosamine, is one of the most abundant natural polysaccharide, second only to cellulose, from which it differs structurally by the presence of acetamide groups. Chitin looks like a white, hard, inelastic and nitrogenous material, widely available in nature, being the major structural component of arthropods’ exoskeleton, mollusk shells, and the cell wall of fungi and yeasts^17–20^. The main source for the industrial production of chitin is waste from the fishing industry, such as crab, shrimp, prawn and lobster shells, with a chitin content ranging from 15 to 40%^17,21,22^. However, the growing market demand for chitin has made it necessary to explore new alternative sources, including fungi and insects. Insects show great potential as a sustainable and readily available alternative source of chitin, which represents up to 25–60% of the dry weight of their cuticle^23–30^. Due to its crystalline structure, which gives it a high hydrophobicity, chitin is insoluble in water, organic and inorganic solvents, and common acidic or basic solutions^31,32^. This poor solubility negatively affects its processing and application, limiting the production of chitin-based products^20^. To broaden its range of application, chitin is deacetylated into chitosan, its more soluble derivative. Due to their properties, like biodegradability, biocompatibility, non-toxicity, adsorption, antioxidant, humectant and antimicrobial activity^18,33,34^, chitin, chitosan and their derivatives are used in industrial and biomedical applications, such as agriculture, food and nutrition, tissue engineering, wastewater treatment, drug delivery, wound healing and cosmetics^19,35–39^. Structurally, chitosan is a cationic polysaccharide consisting of D-glucosamine and N-acetyl D-glucosamine units. Its chemical-physical properties, as well as its applicability, depend mainly on its degree of deacetylation (DD), molecular weight (MW) and amino groups (NH_2_) presence^40^. The free amino groups are important for the polymer polarity. As a result of the protonation of these groups (NH_3_^+^) at pH values below 6.5, chitosan becomes soluble^31,32,40^. Due to their positive charge, these functionally active amino groups are also responsible for the antibacterial and antifungal activity of chitosan, making it interesting for biomedical applications^41^ (Fig. 1).

Chitosan can inhibit the proliferation of many bacteria, fungi and yeasts, with different mechanisms, not all fully clarified^42–45^. The simplest mechanism of action involves electrostatic interactions between the NH_3_^+^ sites of chitosan (positively charged) and the membranes of microbial cells (negatively charged). The interaction alters the permeability of the microbial cell, causing the release of intracellular material^46^. *Chung *et al*.*^47^ have shown the disruption of cell structure of *Escherichia coli* and *Staphylococcus aureus* due to the binding of chitosan to microbial enzymes and nucleotides. The effectiveness of chitosan in altering the amount of calcium on cell walls was also demonstrated^48^. In this case, chitosan exerts its antimicrobial effect by interacting with the stability of peptidoglycan and changing the osmotic balance of the membrane wall^49^. Chitosan can also compromise the energy stability of the membrane by interfering with the electron transport chain and oxygen reduction processes^50^. Another proposed mechanism is related to the chitosan ability to chelate metal ions^51^: by sequestering iron, zinc, copper, cadmium, magnesium and other bivalent cations, it induces damage to the microbial membrane integrity^52^. Positively charged chitosan can also act blocking RNA and protein synthesis, thus inhibiting bacterial growth^53,54^. However, this mechanism requires a reduction in the size of chitosan to allow the penetration into the cellular system^46^. Chitosan, in its polycationic form, shows antimicrobial activity against both Gram-positive and Gram-negative bacteria, acting differently depending on the respective cell membrane structure. In Gram-negative bacteria, chitosan interacts with anionic structures present on their surface, such as lipopolysaccharides and proteins^55^; in Gram-positive bacteria, on the other hand, the polymer interacts directly with their cell wall layer, consisting of negatively charges of peptidoglycan and teichoic acids^56^. No evidence on which bacteria is more effective is reported. Indeed, some studies^57,58^ described a stronger bactericidal action on Gram-negative bacteria, while other authors demonstrated a more powerful activity on Gram-positive bacteria^59,60^.

The antimicrobial activity of chitosan, as well as other properties, also depends on its chemical characteristics (MW and DD) and on some experimental conditions, such as temperature and pH.

Among these features, MW is probably the most related to the antimicrobial activity. In some cases, chitosan with low MW had a greater effect against Gram-negative bacteria, while chitosan with high MW showed a stronger action against Gram-positive bacteria^61^. It has been hypothesized that low MW chitosan can easily cross the cell wall of Gram-negative bacteria, while high MW chitosan acts as a barrier interfering with the proper absorption of nutrients by the microbial cell^62^. But this is not a general consideration, as, in other studies, higher antimicrobial activity against Gram-positive bacteria was found for low MW chitosan^49^. Therefore, also the correlation between the chitosan MW and its antimicrobial activity is still to be better clarified.

The aim of the present work is to investigate the antimicrobial activity of bleached and unbleached chitosan produced from the biomasses of the insect *H. illucens*, such as dead adult flies, pupal exuviae, and larvae exceeding the required production for feed purpose. The antimicrobial tests (diffusion test and microdilution assay) were carried out on *E. coli* (Gram-negative) and *Micrococcus flavus* (Gram-positive). *E. coli*, belonging to the Enterobacteriaceae family, is the etiological agent of some serious human infections, while *M. flavus* (Micrococcaceae) is a saprophyte opportunistic pathogen. Some *Micrococcus* spp. were responsible for infections, such as septic arthritis, prosthetic valve endocarditis, recurrent bacteremia, but also pneumonia in oncological subjects and skin infections in immuno-compromised patients^63^.

## Results and discussion

### Characterization of Chitosan by FTIR

**Figure 2:**
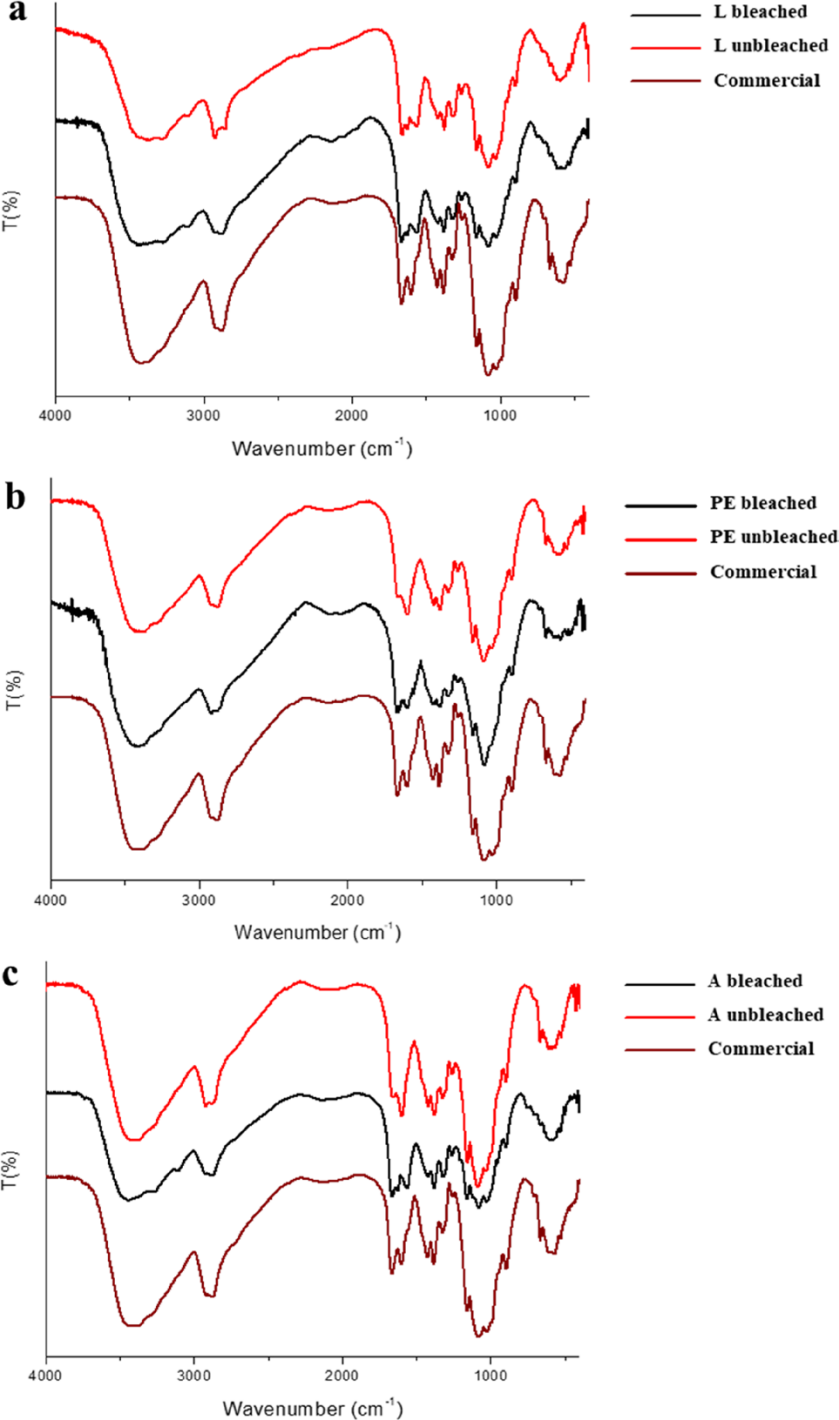
FTIR spectra of bleached (black line) and unbleached (red line) chitosan samples extracted from *H. illucens* larvae (**a**), pupal exuviae (**b**) and dead adults (**c**). Commercial chitosan (wine lines) derived from crustaceans are also reported.

Spectra resulting from FTIR analysis of chitosan samples from *H. illucens* larvae, pupal exuviae and dead adults are shown in Fig. 2 (a-b-c), in comparison with the commercial one. The chitosan identity was confirmed by the presence of characteristic peaks, particularly amide I and amide II bands around 1590 and 1650 cm^-1^, respectively^64–67^. From the spectra no significant differences were found between the bleached and the respective unbleached chitosan, and between them and the commercial one.

### Antimicrobial activity of chitosan samples from *H. illucens*

#### Agar diffusion test

Results of the agar diffusion assay performed on *E. coli* and *M. flavus* are reported in Table 1.

**Table 1:** Diameters (mm) of inhibition zones formed by bleached and unbleached chitosan samples produced from *H. illucens* larvae (L), pupal exuviae (PE) and dead adults (A), commercial chitosan and acetic acid at five different concentrations (1.25, 0.6, 0.3 and 0.15 mg/ml) on *E. coli* and *M. flavus*. Distilled water was tested as negative control. Results are expressed as mean ± standard deviation of diameters measured with agar diffusion test of three independent biological replicates. Different letters indicate significant differences (*p* < 0.05) among treatments for the same concentration (capital letters) and among different concentrations in the same treatment (lowercase letters). The intensity of the antibacterial activity of each sample was evaluated according to the scale proposed by El-masry et al.^63^ and expressed with different symbols depending on the diameter of the respective inhibition zone: +  +  + (inhibition zone > 12 mm, high activity), +  + (inhibition zone 9–12 mm, moderate activity), + (inhibition zone 6–9 mm, slight activity).

Bacterial species	Sample	1.25 mg/ml	0.6 mg/ml	0.3 mg/ml	0.15 mg/ml	1.25 mg/ml	0.6 mg/ml	0.3 mg/ml	0.15 mg/ml
*E. coli*	L bleached chitosan	9 ± 0.5^a A^	9 ± 0.8^a A^	8 ± 0.5^ab A^	7 ± 0.5^b AB^	++	++	+	+
	PE bleached chitosan	9 ± 0.4^a A^	9 ± 0.4^a A^	9 ± 0.5^a AB^	8 ± 0.5^a A^	++	+ +	+ +	+
	A bleached chitosan	10 ± 0.5^a A^	10 ± 0.8^a A^	10 ± 0.8^a BD^	8 ± 0.5^b A^	++	+ +	+ +	+
	L unbleached chitosan	9 ± 0.5^a A^	9 ± 0.2^a A^	8 ± 0.3^ab AC^	7 ± 0.5^b AB^	+ +	+ +	+	+
	PE unbleached chitosan	9 ± 0.3^a A^	9 ± 0.5^a A^	9 ± 0.3^a ACD^	8 ± 0.2^b A^	+ +	+ +	+ +	+
	A unbleached chitosan	10 ± 0.3^a A^	10 ± 0.5^a A^	10 ± 0.7^a BD^	8 ± 0.2^b A^	+ +	+ +	+ +	+
	Commercial chitosan	6 ± 0.5^a B^	6 ± 0.8^a B^	6 ± 0.8^a E^	6 ± 1^a AB^	+	+	+	+
	Acetic acid	–	–	–	–	–	–	–	–
	Distilled water	–	–	–	–	–	–	–	–
*M. flavus*	L bleached chitosan	7 ± 0.3^a A^	7 ± 0.5^a A^	7 ± 0.5^a A^	6 ± 0.5^a A^	+	+	+	+
	PE bleached chitosan	10 ± 0.4^a B^	10 ± 0.4^a B^	10 ± 0.5^a B^	7 ± 0.4^b AC^	++	+ +	+ +	+
	A bleached chitosan	10 ± 0.5^a B^	10 ± 0.7^a B^	10 ± 0.7^a B^	8 ± 0.5^b BC^	++	+ +	+ +	+
	L unbleached chitosan	7 ± 0.3^a A^	7 ± 0.7^a A^	7 ± 0.5^a A^	6 ± 0.2^a A^	+	+	+	+
	PE unbleached chitosan	9 ± 0.8^a B^	8 ± 0.5^ab A^	7 ± 0.5^b A^	7 ± 0.8^b AC^	+ +	+	+	+
	A unbleached chitosan	10 ± 0.2^a B^	10 ± 0.7^a B^	10 ± 0.5^a B^	8 ± 0.2^b BC^	+ +	+ +	+ +	+
	Commercial chitosan	7 ± 0.5^a A^	7 ± 0.5^a A^	7 ± 0.5^a A^	6 ± 0.5^a A^	+	+	+	+
	Acetic acid	–	–	–	–	–	–	–	–
	Distilled water	–	–	–	–	–	–	–	–

All chitosan samples produced from *H. illucens,* as well as the commercial chitosan used as positive control, induced the formation of a measurable inhibition zone against both bacterial species at all the tested concentrations. As expected, distilled water, used as negative control, showed no inhibition zones, whereas acetic acid, as control, had a slight inhibitory effect, with the formation of undefined and therefore unmeasurable inhibition zones, confirming that the observed antimicrobial activity is not due to the acetic acid (the solvent), but effectively to the chitosan samples (Table 1).

**Figure 3:**
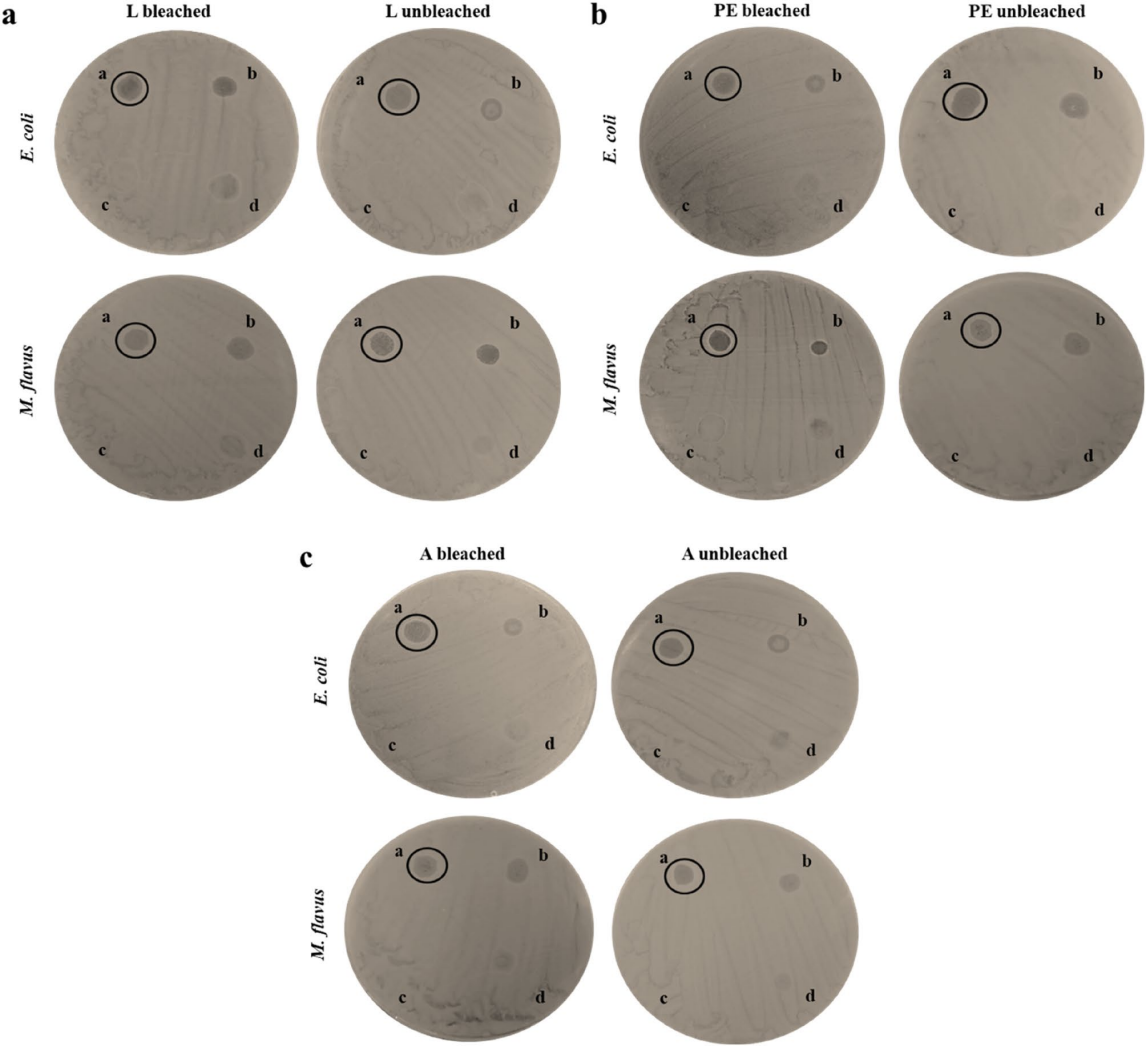
Inhibition zones of bleached and unbleached chitosan samples produced from *H. illucens* larvae (**a**), pupal exuviae (**b**), dead adults (**c**). Chitosan samples from *H. illucens* (*circle a*), commercial chitosan (*circle* *b*), distilled water (*circle* *c*) and acetic acid (*circle* *d*) on *E. coli* and *M. flavus* resulting from the agar diffusion test are reported.

Literature data showed the antimicrobial potential of chitosan, but there was still little information obtained from the investigation of insect-derived chitosan. Moreover, it is still unknown whether the biopolymer acted better on Gram-positive or Gram-negative bacteria. In this work, for the first time, the antimicrobial potential of chitosan produced from different biomasses of *H. illucens* (larvae, pupal exuviae and dead adults) is investigated. Pictures of the inhibition zones of all bleached and unbleached chitosan samples, acetic acid and distilled water were reported in Fig. 3. Our data can be compared with those reported by Lagat et al*.*^68^ and Kemboi et al*.*^69^. Lagat et al*.*^68^ tested chitosan from pupal exuviae of *H. illucens* against two Gram-negative bacteria (*E. coli* and *Pseudomonas aeruginosa*) and two Gram-positive bacteria (*S. aureus* and *Bacillus subtilis*), at the concentrations of 5, 2.5, 1 and 0.5 g/ml. Kemboi et al*.*^69^ tested chitosan from pupal exuviae of *Hermetia illucens* on *Ralstonia Solanacearum,* using disc diffusion test at the similar concentration range. Both obtained larger inhibition diameters than ours, but at much higher concentrations. Indeed, our results, considering the different order of magnitude of the concentrations tested, revealed a good antimicrobial capacity and were very encouraging.

Basseri et al.^70^ tested chitosan obtained from different developmental stages (adults and nymphs) of two cockroaches, *Periplaneta americana* and *Blattella germanica*. They tested solutions of chitosan at the concentration of 10 mg/ml on three different bacteria: *E. coli* and *P. aeruginosa*, as Gram-negative, and *S. aureus,* as Gram-positive species*.* Despite the tested concentration, which was eight times higher than ours, the inhibition diameters (ranging between 7–10 mm) obtained by Basseri et al*.*^70^ were comparable to those obtained in this work. As in Lagat et al*.*^68^, chitosan from the three biomasses of *H. illucens* are effective. The results obtained are also within the ranges identified by Kaya et al*.*^71^ who investigated the antimicrobial activity of chitosan from the myriapod *Julus terrestris* on some Gram-positive spp. Furthermore, all chitosan from *H. illucens* was found to be more effective, with larger inhibition diameters than those obtained from chitosan of *Tenebrio molitor, and Zophobas morio* (1–2 mm) against Gram-negative (*E. coli* and *S. aureus*) and Gram-positive (*Bacillus cereus* and *Listeria monocytogenes*)^72^ bacteria.

The results obtained in this study on the antibacterial activity of chitosan derived from each of the three biomasses of *H. illucens* were also comparable to those obtained from chitosan derived from crustaceans, the most widely used commercial source^73,74^. Particularly, Aliasghari et al*.*^74^ studied the activity of the biopolymer on different *Streptococci* spp. (Gram-positive bacteria), testing concentrations ranging from 5 to 1.25 mg/ml. At the concentration of 1.25 mg/ml, they obtained diameters in the range of 8.50–9 mm, comparable to those obtained by our chitosan against the Gram-positive *M. flavus*, at the same chitosan concentration. Therefore, chitosan derived from insects, and in particular from *H. illucens*, is a valid alternative to commercial chitosan for antimicrobial activity.

In order to evaluate the intensity of the antimicrobial activity of chitosan samples, the scale proposed by El-masry et al*.*^75^ was adopted. The activity of chitosan against *E. coli* and *M. flavus* was thus classified as high, moderate or slight, depending on the diameter of the respective induced inhibition zone (Table 1). Most of the tested samples had a moderate or high antimicrobial activity against both bacterial species. Only at the lowest concentration (0.15 mg/ml), all chitosan samples were slightly active. All bleached and unbleached samples, produced from *H. illucens* had higher antimicrobial activity than the commercial one, being moderately active at almost all concentrations (Table 1). A slight activity was found for bleached and unbleached chitosan from larvae against *E. coli* at 0.3 mg/ml, and against *M. flavus* in the range 1.25–0.3 mg/ml. For unbleached chitosan from pupal exuviae a low antimicrobial activity was detected on *M. flavus* in the range 0.6–0.15 mg/ml. A high antimicrobial activity was performed only by chitosan from adults against *E. coli* and by chitosan from pupal exuviae against *M. flavus*, both at the highest concentration (Table 1). These results confirmed the antimicrobial potential of bleached and unbleached chitosan, produced from different samples of *H. illucens,* tested against *E. coli* and *M. flavus*. The analysis of inhibition diameters showed no difference in the effect of insect chitosan against Gram-positive or Gram-negative bacteria.

#### Determination of the minimum inhibitory concentration (MIC) through the microdilution assay

**Figure 4:**
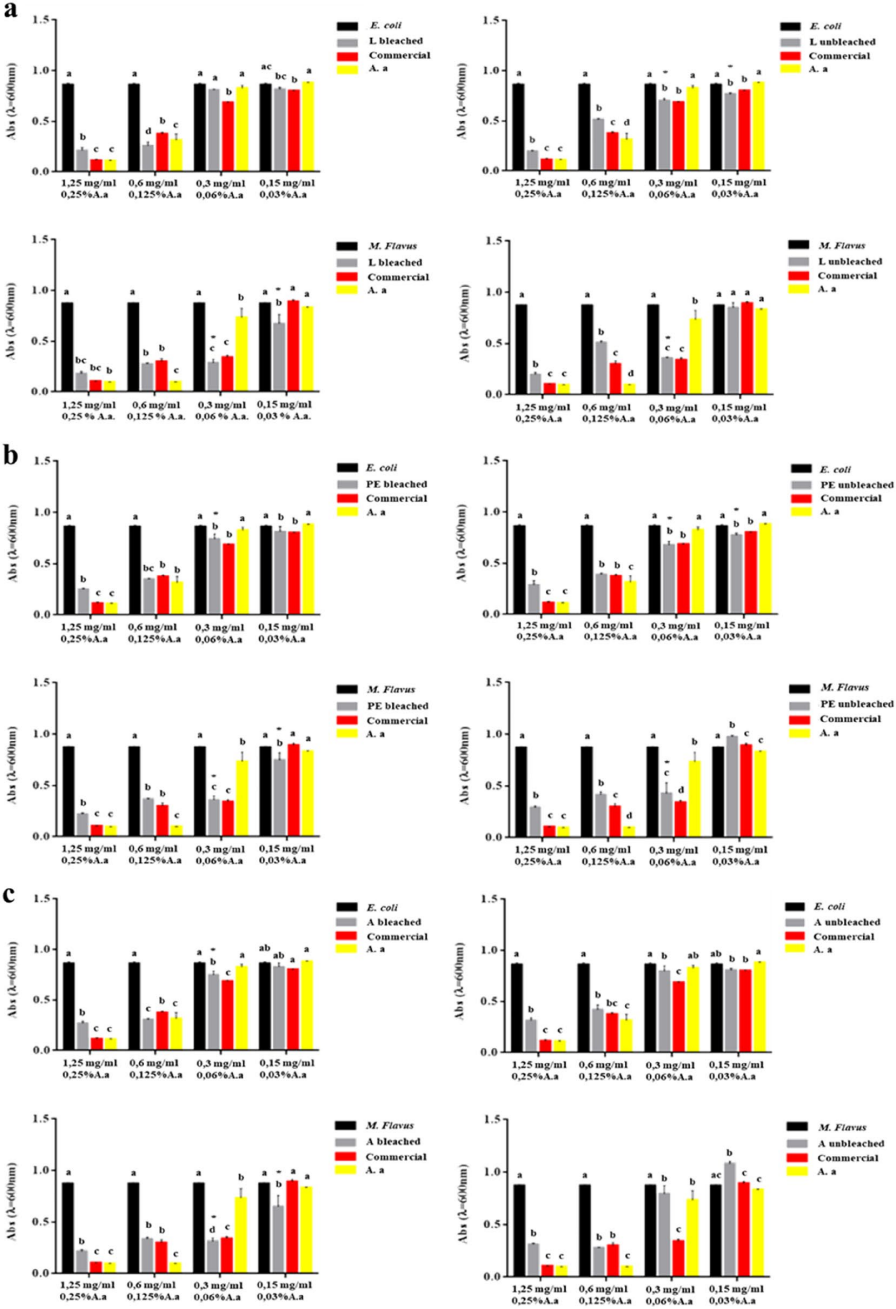
Results of microdilution assay for bleached and unbleached chitosan from larvae (**a**), pupal exuviae (**b**) and dead adults (**c**) of *H. illucens*, commercial chitosan and acetic acid at the four concentrations of 1.25, 0.6, 0.3, 0.15 mg/ml against *E. coli* and *M. flavus*. Bars indicate the absorbance of the bacterial culture (black bars) and that of the culture treated with *H. illucens* chitosan samples (gray bars), commercial chitosan (red bars) and acetic acid (yellow bars). Data are presented as mean ± standard error of three independent experimental biological replicates. Different letters indicate significant differences (*p* < 0.05) between absorbance values of the bacterial culture alone and that of bacteria treated with the different concentrations of each treatment. Asterisks indicate significant differences (*p* < 0.05) among treatments for the same concentration. Data are analyzed with two-way ANOVA and Bonferroni post-hoc test.

Results of microdilution assay for unbleached and bleached chitosan derived from *H. illucens* larvae, pupal exuviae and dead adults were reported in Fig. 4(a-b-c). Six chitosan samples were tested at four concentrations of 1.25, 0.6, 0.3 and 0.15 mg/ml and for each chitosan the MIC value was determined. At the highest concentrations, 1.25 and 0.6 mg/ml, all insect chitosan samples significatively inhibited the growth of *E. coli* culture as much as the commercial one (*p* < 0.05). However, the inhibitory effect cannot be attributed to chitosan alone, since at these concentrations even acetic acid, its solvent, had antimicrobial power. The same result was obtained against *M. flavus* for the same sample concentrations. Bleached chitosan from larvae showed no inhibition of *E. coli* culture, while its activity against *M. flavus* was statistically significant at 0.3 and 0.15 mg/ml, attributing the MIC value to the latter concentration. On the other hand, the unbleached chitosan showed antimicrobial activity on both *E. coli* and *M. flavus*. Particularly, on *E. coli*, the activity was shown both at 0.3 mg/ml and 0.15 mg/ml, while on *M. flavus* the sample lost its activity at 0.15 mg/ml, so the MIC value was 0.3 mg/ml (Fig. 4a). Bleached chitosan from pupal exuviae showed antimicrobial activity at 0.3 mg/ml, tested on *E. coli*, while on *M. flavus* the value of MIC was 0.15 mg/ml. In the case of unbleached pupal exuviae chitosan samples, the MIC value detectable on *E. coli* and *M. flavus* was 0.15 mg/ml and 0.3 mg/ml, respectively (Fig. 4b). At the concentration of 0.3 mg/ml, bleached chitosan from adults had good antimicrobial activity on *E. coli*, which was statistically significant compared to acetic acid alone. The same sample, on *M. flavus* showed a significant effect compared to the acetic acid at 0.15 mg/ml. For unbleached adult chitosan, there was no good antimicrobial activity attributable to chitosan alone on both *E. coli* and *M. flavus* (Fig. 4c).

The main problem for antimicrobial activity evaluation of chitosan was to find the concentration at which acetic acid had no longer effect, and the inhibiting activity can be ascribed exclusively to chitosan. The concentration of 0.15 mg/ml for some of the samples was too low to achieve antimicrobial effect. On the other hand, at concentrations above 0.3 mg/ml, acetic acid probably exerted a greater inhibitory effect, covering that of chitosan. As reported by Liu et al*.*^76^, acetic acid appeared to lose its antimicrobial capacity at concentrations below 200 ppm, equivalent to 0.04% acetic acid in solution. Therefore, the carried-out experiments showed that the bacterial growth-inhibiting concentration of chitosan was less than or equal to 0.3 mg/ml, the concentration at which acetic acid (0.06%) would seem to lose its antibacterial activity.

For bleached and unbleached chitosan samples from *H. illucens*, identified MIC values are reported in Table 2. The obtained results showed that the lowest MIC values (0.15 mg/ml) on *E. coli* was obtained by chitosan from unbleached larvae and pupal exuviae, while on *M. flavus* they were found for all three bleached biomasses.

**Table 2:** MIC values of bleached and unbleached chitosan samples produced from *H. illucens* larvae (L), pupal exuviae (PE) and dead adults (A).

Bacterial species	Sample concentration				
	Chitosan sample	1.25 mg/ml	0.6 mg/ml	0.3 mg/ml	0.15 mg/ml
*E. coli*	L bleached				
	PE bleached			*	
	A bleached			*	
	L unbleached				*
	PE unbleached				*
	A unbleached				
*M. flavus*	L bleached				*
	PE bleached				*
	A bleached				*
	L unbleached			*	
	PE unbleached			*	
	A unbleached				

Chitosan alone has a slight ability to reduce the growth of microorganisms^77^. Most studies report the use of modified chitosan with other functional groups, like hydrolyzed starch/chitosan, based Schiff bases of chitosan or quaternary N-alkyl and N, N-dialkyl chitosan derivatives, in order to improve its antibacterial properties^78–80^. The present work is the first one investigating the antimicrobial potential of chitosan from three biomasses of *H. illucens* (larvae, pupal exuviae and dead adults) both through a qualitative evaluation with the agar diffusion test and a quantitative evaluation with the microdilution assay, for the final determination of the MIC. Indeed, according to Ramasamy et al.^81^, MIC tests enhanced the value of data obtained from agar diffusion tests. Due to the different methodologies used, the MIC values determined are often controversial and unknown; indeed, in many studies, the MIC value was determined only through a qualitative evaluation of the turbidity degree of the tubes containing the chitosan sample and the culture on which it was tested^81,82^. Thus, a direct comparison with the literature was not always easy.

A comparison was possible with the work of Lin et al*.*^83^, that investigated the MIC value of pupal exuviae chitosan from *H. illucens* on *P. aeruginosa* and *S. aureus;* the MIC values of our bleached and unbleached chitosan were lower on Gram-positive species (0.15 and 0.3 mg/ml vs 0.6 mg/ml), while they were higher on the Gram-negative species (0.3 and 0.15 mg/ml vs 0.04 mg/ml), respectively. Khayrova et al*.*^84^ also tested the antimicrobial activity of larvae chitosan from *H. illucens* on *E. coli* and *Staphylococcus epidermidis*. The MIC values of our unbleached larvae chitosan were lower on *E. coli* (0.15 mg/ml vs 0.5 mg/ml), while they were slightly higher on Gram-positive species (0.3–0.15 mg/ml vs 0.125–0.0625 mg/ml). On Gram-positive bacteria, using the same method, MIC values for all our chitosan samples were better than those obtained by Aliasghari et al*.*^74^ for chitosan from crustaceans (0.15 and 0.3 mg/ml vs 1.25 and 2.5 mg/ml). Our results were also better than those reported by Li et al.^85^ who tested crustacean chitosan on both species of bacteria (0.15 and 0.3 mg/ml vs 0.625 mg/ml on Gram-negative and 0.15 and 0.3 mg/ml vs 0.313 mg/ml on Gram-positive, respectively).

Other studies related to chitosan from crustaceans and fungi tested against Gram-positive bacteria, reported MIC values higher than those obtained in the present work^86,87^. Therefore, chitosan from *H. illucens* biomasses may have a stronger inhibiting activity, as a lower concentration was sufficient to exert the effect.

Several observations can be deduced from the results obtained. As previously reported, *E. coli* and *M. flavus*, as Gram-negative and Gram-positive bacteria respectively, differ in the structure of their bacterial wall; chitosan, positively charged, interacts with the negative charges of the bacteria with a different mechanism depending on the strain. Despite these differences, it was possible to demonstrate how chitosan from *H. illucens* is active against both Gram-negative and Gram-positive bacteria. This allowed us to deduce how the antimicrobial activity of chitosan from the three biomasses of *H. illucens*, is mostly related to its chemical-physical and morphological properties, beyond its mechanism of action. This hypothesis is also demonstrated by the inhibition zones obtained from the agar diffusion tests. Looking forward, the obtained results make our biopolymer particularly versatile and therefore suitable for future new applications.

Broth microdilution assays confirmed the good antimicrobial activity of chitosan from *H. illucens*, with rather low MIC values ranging from 0.3 to 0.15 mg/ml. For some chitosan samples from larvae and adults it was not possible to identify the MIC value on one or both strains; however, it was always possible to determine this value for chitosan samples from pupal exuviae, which proved to be the biomass of choice. This could be attributed to the different nature of the starting biomass for chitosan production, to the possible presence of impurities in the final chitosan samples, but also to the extraction process, particularly the bleaching step. Indeed, for unbleached adult chitosan from *H. illucens* it was not possible to determine this value neither on *E. coli* nor *M. flavus*, probably due to the pigments that hide some positive charges of chitosan, resulting in a lower general antimicrobial activity. In contrast, for bleached larvae chitosan, the presence or absence of pigments may be a discriminating factor in determining the MIC value. In general, we can deduce how the bleaching step influences the specific chemical-physical and morphological characteristics of the chitosan samples and therefore also its antimicrobial activity that will be expressed with different intensity between the two tested bacterial strains. Indeed, it was generally observed that on *E. coli* the unbleached chitosan samples show lower MIC values than the bleached ones (0.15 mg/ml vs 0.3 mg/ml), exactly the opposite on *M. flavus*, with better MIC values for the bleached samples.

This study provided the value of insects, and in particular of *H. illucens,* as an alternative source of chitin and chitosan to crustaceans, also in antimicrobial applications.

## Conclusions

Currently, the production of chitin and chitosan from insects is only carried out on a laboratory scale, using the same procedures as for crustaceans, the common commercial source. In recent years, bioconverter insect farms are becoming widely used. In particular, *H. illucens* fits perfectly into a zero-waste circular economy system for organic waste management. Exceeding larvae, primarily used for protein feed production, in addition to pupal exuviae and dead adults, the only waste products of the insect farm, can be recovered and used for the extraction and production of chitin and chitosan. It is known that chitosan has antimicrobial activity, but there are still few studies investigating this property of chitosan from insects, and even fewer from the bioconverter *H. illucens*. Moreover, in the works examining the antimicrobial activity of the polymer, only the bacterial culture on which the polymer acts is considered as control, without investigating the real activity of the solvent alone, acetic acid. It can therefore be deduced how the solvent effects, in itself antibacterial, may have influenced the activity of chitosan tested in other papers. The present work is the first investigation on the antibacterial potential of chitosan produced from different biomasses of the dipteran *H. illucens*. All the tested chitosan samples (larvae, pupal exuviae and dead adults) induced the formation of inhibition zones against *E. coli* and *M. flavus*, suggesting a good antimicrobial ability both against Gram-negative and Gram-positive bacteria species. Good antibacterial activity of all chitosan samples on *E. coli* and *M. flavus* was also confirmed by quantitative analysis performed with microdilution assays. In particular, both bleached and unbleached pupal exuviae of *H. illucens* always showed significant antimicrobial activity on both *E. coli* and *M. flavus*. This property seems relevant in view of biomedical and pharmaceutical applications. Furthermore, this confirms the validity of insects, more specifically of *H. illucens*, as an alternative source to crustaceans to extract and use chitin and chitosan.

## Material and methods

### Insect sample preparation

Insect samples of whole larvae, pupal exuviae and dead adults were provided by Xflies s.r.l (Potenza, Italy). Raw insects were dried in oven (Conlabo s.r.l., Potenza, Italy) at 60 °C for 48 h and then ground into powder using a laboratory blender (Waring Commercial Stamford, USA). The powdered samples were subjected to the chitin extraction process. Commercial chitosan derived from crustacean shells, used as control, were purchased from Sigma-Aldrich (St. Louis, Missouri, USA).

### Chitin and chitosan production

Chitin extraction process and deacetylation in chitosan was performed as described by Triunfo et al*.*^88^. Briefly, insect samples were firstly demineralized using 0.5 M CH_2_O_2_ (Sigma-Aldrich St. Louis, Missouri, USA) for 1 h at room temperature, under stirring. After washing with distilled water to restore a neutral pH, the demineralized biomass was stirred with 2 M NaOH (Sigma-Aldrich St. Louis, Missouri, USA) for 2 h at 80 °C to remove proteins. After the deproteinization process, chitin can be subjected to a bleaching procedure using 5% H_2_O_2_ (Sigma-Aldrich St. Louis, Missouri, USA) for 1 h at 90 °C, under stirring. After washing, bleached chitin was dried in oven at 60 °C and subject to heterogeneous deacetylation. Heterogeneous deacetylation was performed by incubating chitin (bleached or unbleached) with 12 M NaOH (Sigma-Aldrich St. Louis, Missouri, USA) for 4 h at 100 °C, under stirring. The deacetylated chitin was then washed thoroughly with distilled water until the reaching of a neutral pH. According to Hahn et al*.*^22^, the deacetylated material was further purified by incubation with 1% acetic acid (Sigma-Aldrich St. Louis, Missouri, USA). The obtained chitosan was washed with distilled water to restore neutrality and lyophilized.

### Fourier-transformed infrared spectroscopy (FTIR)

IR spectra of the bleached and unbleached chitosan samples were recorded using a Jasco 460Plus IR spectrometer. They were scanned with a resolution of 4 cm^-1^ and 100 accumulations and the transmittance (T%) was evaluated in the range of wavelength 4000 – 400 cm^-1^. All chitosan samples, previously dried and pulverized, were mixed with KBr and pressed in order to obtain 1 cm diameter tablets. The resulting spectra were processed using JASCO Spectra Manager software.

### Antimicrobial assays

#### Preparation of chitosan solutions

Stock solutions of bleached and unbleached chitosan of 5 mg/ml from *H. illucens* samples (larvae, pupal exuviae and dead adults), and commercial chitosan were dissolved in 1% acetic acid. After stirring, the solutions were filtered (0.45 µm filter) to remove contaminants and stored at 4 °C. Acetic acid itself has a good antimicrobial activity, depending on the concentration and for this reason, in this work, we tested also acetic acid alone as control^89,90^. Particularly, for agar diffusion tests, serial dilutions were made, obtaining concentrations including 2.5, 1.25, 0.6, 0.3 and 0.15 mg/ml of chitosan (both from *H. illucens* and commercial). The respective serial dilutions were also made for acetic acid, obtaining concentrations of 0.5%, 0.25%, 0.125%, 0.06% and 0.03%. The range between 1.25 and 0.15 mg/ml was applied to test the antimicrobial activity of *H. illucens* chitosan samples, according to Kaya et al*.*^27^. The highest concentrations (5 and 2.5 mg/ml) were excluded as it was not possible to discriminate the antimicrobial activity of commercial chitosan from its solvent, acetic acid (data not shown).

#### Preparation of the microbial suspension

Bacteria (a colony of *E. coli* and *M. flavus*, respectively), were inoculated into 10 ml of sterile Luria Bertani (LB) culture medium, prepared with 1% of tryptone (Sigma-Aldrich St. Louis, Missouri, USA), 0.5% of yeast extract (Sigma-Aldrich St. Louis, Missouri, USA) and 0.5% of sodium chloride (Sigma-Aldrich St. Louis, Missouri, USA), and were placed in a water bath shaker at 37 °C, at 150 rpm for 18 h.

#### Agar diffusion test

Agar diffusion test was employed for evaluating the antimicrobial activity of the different chitosan samples. Bacteria (*E. coli* and *M. flavus*) were homogeneously distributed on sterile Petri dishes containing 25 ml of previously solidified LB-Agar medium (LB with 1.5% bacteriological Agar (Sigma-Aldrich St. Louis, Missouri, USA)), using a cotton swab. Afterwards, 5 μl of each chitosan concentration (1.25, 0.6, 0.3 and 0.15 mg/ml) was spotted onto the LB agar plates and incubated at 37 °C for 24 h. For each plate, acetic acid and solution of commercial chitosan were used at same concentrations as positive controls, while distilled water as a negative control. The diameter of the inhibition zones (mm) was used to evaluate the antimicrobial activity. Results were expressed as mean ± standard deviations of three independent biological replicates.

#### Evaluation of the minimum inhibitory concentration (MIC) by the microdilution assay

A microdilution assay was firstly carried out on both, *E. coli* and *M. flavus,* using commercial chitosan, in order to identify the range of concentrations (mg/ml) at which it exerts its antibacterial action (data not shown). Serial dilutions in 96 wells plates were made for all chitosan samples, at the following concentrations: 1.25, 0.6, 0.3 and 0.15 mg/ml. Solutions of acetic acid alone were tested at the same concentrations (corresponding to 0.25, 0.125, 0.03 and 0.015%, respectively) and each bacterial culture (*E. coli* and *M. flavus)* was also used as a negative control. The bacterial culture was used at a concentration of 10^6^ CFUs/ml for both species. The 96 well plates were then incubated at 37 °C for 24 h, and bacterial concentrations, measured as absorbance at a wavelength of 600 nm, were then evaluated using a Multiskan Go spectrophotometer (Thermo Scientific, Waltham, MA, USA). The minimum inhibitory concentration (MIC) was determined as the lowest concentration of chitosan samples for which no bacterial growth was observed.

### Statistical analysis

All experiments were performed in triplicates and results of agar diffusion and microdilution assays were expressed as mean ± standard deviation. Data of the MIC determination were analyzed with Two-way ANOVA, with Bonferroni *post-hoc* test. All statistical analyses were performed using GraphPad Prism version 6.01 for Windows (GraphPad Software, La Jolla, California USA—www.graphpad.com).

## Data Availability

The datasets used and/or analysed during the current study are available from the corresponding author on reasonable request.
